# Does glucagon have a positive inotropic effect in the human heart?

**DOI:** 10.1186/s12933-018-0791-z

**Published:** 2018-11-27

**Authors:** Jesus Hernández-Cascales

**Affiliations:** Departamento de Farmacologia, Facultad de Medicina, Universidad de Murcia Facultad de Medicina, Campus de Espinardo, Espinardo, 30100 Murcia, Spain

**Keywords:** Glucagon, Inotropic effect, Heart failure

## Abstract

Glucagon is considered to exert cardiostimulant effects, most notably the enhancement of heart rate and contractility, due to the stimulation of glucagon receptors associated with Gs protein stimulation which causes adenylyl cyclase activation and the consequent increase in 3′,5′-cyclic adenosine monophosphate production in the myocardium. These effects have been extensively demonstrated in experimental studies in different animal species. However, efforts to extrapolate the experimental data to patients with low cardiac output states, such as acute heart failure or cardiogenic shock, have been disappointing. The experimental and clinical data on the cardiac effects of glucagon are described here.

## Introduction

Glucagon is a polypeptide hormone produced and secreted by the alpha cells of the pancreatic islets of Langerhans; it increases glucose production and counteracts the effect of insulin in maintaining normoglycaemia in the fasting state. In addition to its metabolic effects, glucagon is considered to be a cardiostimulant agent that increases heart rate and contractility [1, 2]. The mechanism responsible for these effects is the stimulation of glucagon receptors associated with Gs protein, stimulation which causes adenylyl cyclase activation and the consequent increase in 3′,5′-cyclic adenosine monophosphate (cAMP) production in the myocardium [2]. Glucagon also seems to inhibit the activity of cyclic nucleotide phosphodiesterase enzymes, which breakdown cAMP into its product 5′AMP [2]. Thus, the combination of enhanced cAMP production and the reduction in cAMP hydrolysis leads to an increase in myocardial cAMP levels that is responsible for the cardiac actions of glucagon. Indeed, the chronotropic effect of glucagon is a consequence of the increase in cAMP levels in the sinoatrial node, which is the primary pacemaker of the heart, and the determinant of cardiac automaticity and generation of the heart beat [3]. Elevation in cAMP facilitates its binding to and activation of cyclic nucleotide gated channels, leading to an inward current carried by Na^+^ and K^+^ ions (funny current), which is considered the most important determinant of cardiac automaticity [3]. Likewise, cAMP also regulates the spontaneous, rhythmic sarcoplasmic reticulum Ca^2+^ release, via ryanodine receptors. This is a vital factor in the generation of sinoatrial node rate by activating an inward Na^+^–Ca^2+^ exchange current that accelerates firing of the pacemaker [4].

Cardiac contractility is also regulated by the cAMP-dependent protein kinase A that phosphorylates different substrates, including the L-type Ca^2+^ channel or ryanodine receptors, which are essential for controlling muscle contraction during each heartbeat. An incoming action potential leads to the opening of voltage-dependent L-type Ca^2+^ channels and activation of a relatively small Ca^2+^ influx current; this triggers a quantitatively larger intracellular Ca^2+^ release from sarcoplasmic reticulum Ca^2+^ stores through ryanodine receptors, the final event responsible for cardiac contraction. This process, so-called “Ca^2+^ induced Ca^2+^ release”, is intensified by glucagon-induced cAMP enhancement, leading to an increase in cardiac contractility [5]. The positive inotropic effect of glucagon in the hearts of different animal species was first described in 1960 by Fara and Tuttle [6], and it is also considered to occur in humans [1, 2]. Based upon this hypothesis, glucagon has been given for treating low cardiac output disorders [1, 2, 7], but the inotropic effect of glucagon in the human heart and its beneficial effects when given to these patients have not been proved. Experimental and clinical features of glucagon effects on cardiac contractility are discussed next.

## Animal studies

The earliest report on the inotropic effect of glucagon was presented by Farah and Tuttle [6] and showed an increase in heart rate and contractility in dogs after adding glucagon to heart–lung preparations. When heart failure was induced in these preparations by means of pentobarbital, glucagon caused a recovery to control levels. Glucagon also caused inotropic effects in isolated preparations of different animal species. However, glucagon did not produce any significant changes in heart rate, ECG, or blood pressure after being given intravenously or intracardially to anaesthetized dogs. Further investigations showed both species and regional differences within the myocardium for the inotropic effects of glucagon. For instance, glucagon markedly increased left ventricular pressure, dP/dt, and ventricular cAMP levels of the paced rat heart but had no effect on any of these variables in the paced guinea pig heart [8]. Additionally, isolated auricles of the dog, cat or guinea pig, but not of the rabbit, responded to glucagon by an increase in contractility [6]. The inotropic effects of glucagon seem to be more marked at the ventricular than at the atrial level. For instance, in the dog heart, glucagon produces a robust inotropic effect in ventricular myocardium [9] but only a slight contractile effect in atrial myocardium [10]. Furthermore, glucagon increases contractility in the ventricle, but not in the atrium, of the rat heart [11, 12]. In contrast to the differences reported for the inotropic effects of glucagon, its positive chronotropic effect has been consistently obtained in both “in vivo” and “in vitro” studies with isolated atrias obtained from of dog, cat, guinea-pig and rat hearts [3, 6, 10].

## Clinical data

Soon after experimental studies showed its positive inotropic and chronotropic effects, glucagon was given to patients. In a pioneer study, its cardiovascular effects in humans were investigated during diagnostic cardiac catheterization. In 11 patients, 3–5 mg given intravenously significantly increased cardiac index, mean arterial pressure, heart rate and maximum rate of left ventricular pressure development, but left ventricular end-diastolic pressure and systemic vascular resistance did not change significantly. In another group of six patients receiving a 1 mg bolus of glucagon, cardiac index and systolic ejection fraction rate increased, whereas systemic vascular resistance decreased. Based on these results, the authors propose glucagon as a potentially useful drug for treating acute heart failure [13]. However, conflicting results were obtained when glucagon was given for treating low cardiac output states. For instance, 24 out of 50 patients who had either heart failure, cardiogenic shock or both showed some clinical improvement after adding glucagon to a combined therapy which included digitalization, furosemide and spironolactone. However, the other 26 patients did not respond, and 16 of them died [14]. In another study, a continuous infusion of glucagon at an average dose of 4 mg/h over several days was reported to produce distinct improvement in the clinical state of 12 of 16 patients, with heart failure or cardiogenic shock. This improvement, noted by an increase in blood pressure and urinary output as well as a decrease in dyspnea, pulmonary rales, diaphoresis and peripheral edema, could have been due to the concomitant medication administered since 9 of these patients who were not previously treated with diuretic agents received diuretics in the course of glucagon therapy [15]. Some evidence indicates that the beneficial effect on cardiac performance, reported in some cases after glucagon administration, is more related to the effect on peripheral vascular resistance than to a direct positive inotropic effect of glucagon. For example, intravenous glucagon produced a rise in cardiac output and heart rate in three patients with severe aortic stenosis. Left ventricular end-diastolic pressure rose considerably, and there was a conspicuous increase in left ventricular volume. However, mean circumferential shortening rate, as an index of contractility, was little affected. As left ventricular systolic pressure rose, there was a considerable increase in wall force, possibly due to the Frank-Starling effect in the dilated left ventricle. Thus, the authors concluded that cardiac changes observed were largely secondary to an increase in venous return [16]. In a further study aimed at investigating the central and peripheral vascular haemodynamic effects of glucagon in patients with organic heart disease, 2 or 5 mg glucagon intravenously produced a significant increase in cardiac output and enhanced cardiac performance, but a lowering of peripheral and pulmonary vascular resistances as well as lowering of pulmonary arterial pressure was observed [17]. Therefore, these results suggest that the vasodilatory effects of glucagon [18] decrease systemic vascular resistance [13, 17], reduce cardiac load and improve renal function, thus ameliorating cardiac performance and the clinical state of the patients. The metabolic effects of glucagon may contribute to improve cardiac performance. Indeed, hyperglycaemia, resulting from gluconeogenesis and glycogenolysis in the liver [16], could ameliorate myocardial metabolism and consequently cardiac function, given the “metabolic remodelling” occurring in heart failure that promotes cardiac glucose oxidation in preference over fatty acid oxidation, the major source of energy for myocardium in normal conditions [19]. The possibility that the haemodynamic effects of glucagon reported in humans are due to an increase in cardiac contractility resulting from activation of glucagon receptor is very unlikely since glucagon is devoid of any direct inotropic effect in human heart. This was demonstrated “in vitro” with ventricular papillary muscles obtained from human explanted hearts [20], as well as in trabeculas of atrial myocardium obtained from patients undergoing cardiac surgery for valve repair (unpublished results). This notion is further supported by the recent finding that mRNA transcripts for glucagon receptors are absent from the left ventricle (the most important cardiac chamber in maintaining adequate haemodynamic parameters) and that only traces have been detected in left or right atria and right ventricle in 2 out of 15 human hearts studied [21]. In agreement with this finding, the effect of glucagon on myocardial contractile capability in humans has been reported to be only slight [22] or null [23]. Indeed, although, as indicated above, positive results have been reported in some cases, glucagon is considered devoid of beneficial clinical effects in patients with congestive heart failure, and its administration is not recommended in current heart failure therapeutics guidelines [24–27]. In beta blocker or calcium channel blocker overdose, clinical improvement has been associated with glucagon administration in multiple case reports, but its clinical efficacy has not been assessed in any controlled clinical trial; some of the beneficial effect reported could have been due to other concomitant therapies received by these patients [28]. Indeed, glucagon does not consistently improve survival in these patients, and failure to respond to glucagon, particularly in subjects with propranolol toxicity, has been reported [29]. The beneficial effects reported for glucagon in calcium or β-receptors antagonist overdose seems to be due to reversing bradycardia rather than improving depressed myocardial contractility [29]. Indeed, glucagon produces positive chronotropic effects in humans [9, 13, 17], which could be due to a higher glucagon receptor expression in the sinoatrial node than in working myocardium, similar to rat right atria [3]. Additionally, glucagon-induced increases in sympathetic nerve activation may contribute to the chronotropic effect of glucagon since experimental evidence indicates that glucagon stimulates sympathetic activity, acting at the hypothalamic level [30] and elevating circulating catecholamines [31]. However, further research is necessary to ascertain the actual mechanism responsible for the beneficial effect of glucagon in patients with symptomatic bradycardia.

## Summary

In summary, the available evidence is against a positive inotropic effect of glucagon in the human heart. Thus, it should not be given as an inotropic agent for treating low cardiac output states such as acute heart failure or cardiogenic shock. However, experimental and clinical evidence supports its positive chronotropic effect, which could prove useful for treating symptomatic bradycardia, particularly in cases of calcium or β-receptor antagonist overdose.

## Abbreviations

cAMP: 3′,5′-cyclic adenosine monophospahate
ECG: electrocardiogram
mRNA: messenger ribonucleotide acid
